# Supplementary figures and images for: A computational approach to fighting type 1 diabetes by targeting 2C Coxsackie B virus protein with flavonoids
Source: PLoS One. 2023 Aug 30;18(8):e0290576. doi: 10.1371/journal.pone.0290576 (PMC10468086; doi:10.1371/journal.pone.0290576)

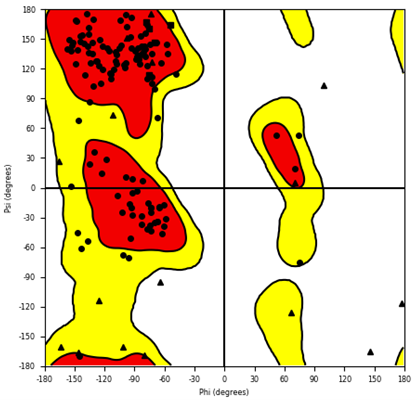

Supplement: S1 Fig — (PNG) [file pone.0290576.s003.png]
