# Supplementary material for: A computational approach to fighting type 1 diabetes by targeting 2C Coxsackie B virus protein with flavonoids
Source: PLoS One. 2023 Aug 30;18(8):e0290576. doi: 10.1371/journal.pone.0290576 (PMC10468086; doi:10.1371/journal.pone.0290576)
Supplement: Attachment — Submitted filename: Comments.docx [file pone.0290576.s004.docx]

The article “Whole genome and RNA sequencing analyses for 254 Taiwanese hepatocellular carcinomas.

This study used whole genome sequencing (WGS), total RNA sequencing (RNA-seq), and clinical data to explore the complex interactions contributing to HCC onset and progression in Taiwan.

Although the study is valuable, it has some shortcomings.

Various situations should be considered that will increase the research value.

Add the significance of your work.

Add limitation of the study

Typos should be corrected. The article should be accepted after Minor revision.
