# Supplementary material for: A computational approach to fighting type 1 diabetes by targeting 2C Coxsackie B virus protein with flavonoids
Source: PLoS One. 2023 Aug 30;18(8):e0290576. doi: 10.1371/journal.pone.0290576 (PMC10468086; doi:10.1371/journal.pone.0290576)
Supplement: Attachment — Submitted filename: The work is focusing on in silicon screening for a flavonoid inhibitor of a coxsackie B virus protein to combat type 1 diabetes.docx [file pone.0290576.s005.docx]

The work is focusing on in silicon screening for a flavonoid inhibitor of a coxsackie B virus protein to combat type 1 diabetes (T1D)

The work is interesting but the preliminary nature of the results and lack of quantitative analysis make it much less interesting. Here are some specific points that the authors should address:

1. The writing could be much more concise and focused. The abstract should be more focus centered around the finding not the introduction about virus and T1D.

2. The material and method section could be significantly improved in that many detailed subsections could be merged into one concise experimental subsection for example, the first section about downloading coordinate from PDB and the preparation and evaluation of the structure for further docking analysis could be combined into “preparation of target structure”.

 3. Also, the criteria why the authors did choose this protein in particular as a target should be further elaborated with references. Also, the authors should explain if there is any reason why “1Z8R” got chosen as a target?.

 4. It should be also describe clearly what did the authors do to minimize and optimize the protein structure. I am very puzzling why did the author exploited computational server like SwissPDB to “optimize” the experimental crystal structure obtained from PDB?

5. The results from the binding site prediction to show all the putative active/allosteric sites should be included as a figure. The validation of the binding prediction is also important and should be added in the paper.

6. The ligand database is not clearly defined. It is unclear what computational or experimental approach the authors exploited to pre-screen the top 37 flavonoids before performing molecular docking with AutoDock Vina. The structures/structural illustration of all the compounds from the flavonoid library used in this work should be included as a supplementary material. Top 37 flavonoids with greatest binding should also be described structurally in the supplementary together with ADMET results.

7. Line 116 “Toxicity Analysis” is a misleading title for a subsection because in fact the toxicity analysis is just part of ADMET analysis. The authors should make it clear.

8. Lead identification section is also not very clear of how did the author choose lead. Is there any particular experiment or analysis the author perform to exclude potentially false hit out from the results apart from looking at the calculated binding energy and the drug-likeness result of ADMET analysis.

9. There is no need to add Ramachandran plot and the structure from PDB in the main figure. Figure 1 could be moved to supplementary information.

10. The structurally optimized model (perhaps superimposed with the PDB data) should be added in the figure and the pre-screening of the library should be at least mentioned. The figure to show binding prediction results should be added together with validation.

  11. Figure 2 there is no detail about bond length in the figure. Also this pocket should be shown together with the full structure.

12.The Figure 3 that showed RMSD from simulations requires a lot of explanation. Is this one time simulation with no repeat? Normally, RMSD from all the repeat of MD simulation should be included in the figure. Also, is there any constraint used in the simulation? The authors should also discuss the fact that ligand RMSD shown in this figures could not displace beyond 1.5 A. Based on the pattern of the chart, it looks like there is a cutoff constraints at approximately 1.5 A?

13. The rest of the figures could be improved. Many of them should be included not as main figures but as supplementary figures.

  14. All structures used in this study including protein, ligands, protein-ligand complexes should be deposited. The topology files used in trajectory analysis from MD simulations should be included as supplementary.
