# Supplementary material for: A computational approach to fighting type 1 diabetes by targeting 2C Coxsackie B virus protein with flavonoids
Source: PLoS One. 2023 Aug 30;18(8):e0290576. doi: 10.1371/journal.pone.0290576 (PMC10468086; doi:10.1371/journal.pone.0290576)
Supplement: Attachment — Submitted filename: Response to Reviewers.docx [file pone.0290576.s006.docx]

Journal Requirements:

When submitting your revision, we need you to address these additional requirements.

1. Please ensure that your manuscript meets PLOS ONE's style requirements, including those for file naming. The PLOS ONE style templates can be found at

https://journals.plos.org/plosone/s/file?id=wjVg/PLOSOne_formatting_sample_main_body.pdf and

<https://journals.plos.org/plosone/s/file?id=ba62/PLOSOne_formatting_sample_title_authors_affiliations.pdf>

**ANSWER: Addressed**

2. We suggest you thoroughly copyedit your manuscript for language usage, spelling, and grammar. If you do not know anyone who can help you do this, you may wish to consider employing a professional scientific editing service.

Whilst you may use any professional scientific editing service of your choice, PLOS has partnered with both American Journal Experts (AJE) and Editage to provide discounted services to PLOS authors. Both organizations have experience helping authors meet PLOS guidelines and can provide language editing, translation, manuscript formatting, and figure formatting to ensure your manuscript meets our submission guidelines. To take advantage of our partnership with AJE, visit the AJE website (http://learn.aje.com/plos/) for a 15% discount off AJE services. To take advantage of our partnership with Editage, visit the Editage website (www.editage.com) and enter referral code PLOSEDIT for a 15% discount off Editage services.  If the PLOS editorial team finds any language issues in text that either AJE or Editage has edited, the service provider will re-edit the text for free.

Upon resubmission, please provide the following:

The name of the colleague or the details of the professional service that edited your manuscript

A copy of your manuscript showing your changes by either highlighting them or using track changes (uploaded as a *supporting information* file)

A clean copy of the edited manuscript (uploaded as the new *manuscript* file).

**ANSWER: Addressed. We copyedit the manuscript for language usage, spelling, and grammar.**

3. Thank you for stating the following financial disclosure:

“Dr. Shahid Ullah designed and supervised the project with Dr. Tianshun Gao's assistance and performed data analysis. Zilong Zheng, Farhan Ullah, Wajeeha Rahman, Dr. Anees Ullah contributed to data analysis. Shahid Ullah wrote the manuscript. All authors reviewed the manuscript.

This research is supported by National Natural Science Foundation of China [32100434] and Research Start-up Fund of the Seventh Affiliated Hospital, Sun Yat-sen University [ZSQYBRJH0020]”

Please state what role the funders took in the study.  If the funders had no role, please state: "The funders had no role in study design, data collection and analysis, decision to publish, or preparation of the manuscript."

If this statement is not correct you must amend it as needed.

Please include this amended Role of Funder statement in your cover letter; we will change the online submission form on your behalf.

**ANSWER:** This research is supported by National Natural Science Foundation of China [32100434] and Research Start-up Fund of the Seventh Affiliated Hospital, Sun Yat-sen University [ZSQYBRJH0020]”

Also The funders had the main role in study design, data collection and analysis, decision to publish, or preparation of the manuscript."

4. In your Data Availability statement, you have not specified where the minimal data set underlying the results described in your manuscript can be found. PLOS defines a study's minimal data set as the underlying data used to reach the conclusions drawn in the manuscript and any additional data required to replicate the reported study findings in their entirety. All PLOS journals require that the minimal data set be made fully available. For more information about our data policy, please see http://journals.plos.org/plosone/s/data-availability.

Upon re-submitting your revised manuscript, please upload your study’s minimal underlying data set as either Supporting Information files or to a stable, public repository and include the relevant URLs, DOIs, or accession numbers within your revised cover letter. For a list of acceptable repositories, please see http://journals.plos.org/plosone/s/data-availability#loc-recommended-repositories. Any potentially identifying patient information must be fully anonymized.

Important: If there are ethical or legal restrictions to sharing your data publicly, please explain these restrictions in detail. Please see our guidelines for more information on what we consider unacceptable restrictions to publicly sharing data: http://journals.plos.org/plosone/s/data-availability#loc-unacceptable-data-access-restrictions. Note that it is not acceptable for the authors to be the sole named individuals responsible for ensuring data access.

We will update your Data Availability statement to reflect the information you provide in your cover letter.

**ANSWER: All the data is available in the main manuscript and in supplementary tables, and will be freely available under journal rule.**

5. Please include a separate caption for each figure in your manuscript.

**ANSWER: Addressed**


[Note: HTML markup is below. Please do not edit.]

Reviewers' comments:

Reviewer's Responses to Questions

**Comments to the Author**

1. Is the manuscript technically sound, and do the data support the conclusions?

The manuscript must describe a technically sound piece of scientific research with data that supports the conclusions. Experiments must have been conducted rigorously, with appropriate controls, replication, and sample sizes. The conclusions must be drawn appropriately based on the data presented.

Reviewer #1: No

Reviewer #2: Partly

2. Has the statistical analysis been performed appropriately and rigorously?

Reviewer #1: N/A

Reviewer #2: No

3. Have the authors made all data underlying the findings in their manuscript fully available?

The [PLOS Data policy](http://www.plosone.org/static/policies.action#sharing) requires authors to make all data underlying the findings described in their manuscript fully available without restriction, with rare exception (please refer to the Data Availability Statement in the manuscript PDF file). The data should be provided as part of the manuscript or its supporting information, or deposited to a public repository. For example, in addition to summary statistics, the data points behind means, medians and variance measures should be available. If there are restrictions on publicly sharing data—e.g. participant privacy or use of data from a third party—those must be specified.

Reviewer #1: Yes

Reviewer #2: No

4. Is the manuscript presented in an intelligible fashion and written in standard English?

PLOS ONE does not copyedit accepted manuscripts, so the language in submitted articles must be clear, correct, and unambiguous. Any typographical or grammatical errors should be corrected at revision, so please note any specific errors here.

Reviewer #1: Yes

Reviewer #2: No

5. Review Comments to the Author

Please use the space provided to explain your answers to the questions above. You may also include additional comments for the author, including concerns about dual publication, research ethics, or publication ethics. (Please upload your review as an attachment if it exceeds 20,000 characters)

Reviewer #1: The article “A computational approach to fighting type 1 diabetes by targeting 2C Coxsackie B virus protein with flavonoids
This study will assist in developing medicine and testing it as a treatment for Diabetes Mellitus Type 1 brought on by Coxsackie B4 viruses by giving them an understanding of chemicals against these viruses. Although the study is valuable, it has some shortcomings.
Various situations should be considered that will increase the research value.
Abs tart should be rewritten with clear objectives and scientific language
Add the significance of your work.
Add limitation of the study
Typos should be corrected. The article should be accepted after Major revision.

**ANSWER: Addressed. Comments addressed and manuscript improved appropriately.**

Reviewer #2: The work is focusing on in silicon screening for a flavonoid inhibitor of a coxsackie B virus protein to combat type 1 diabetes (T1D).

The work is interesting but the preliminary nature of the results and lack of quantitative analysis make it much less interesting. Here are some specific points that the authors should address:

1. The writing could be much more concise and focused. The abstract should be more focus centered around the finding not the introduction about virus and T1D.

**ANSWER: Addressed.**

2. The material and method section could be significantly improved in that many detailed subsections could be merged into one concise experimental subsection for example, the first section about downloading coordinate from PDB and the preparation and evaluation of the structure for further docking analysis could be combined into “preparation of target structure”.
**ANSWER: Addressed.**

3. Also, the criteria why the authors did choose this protein in particular as a target should be further elaborated with references. Also, the authors should explain if there is any reason why “1Z8R” got chosen as a target?.

**ANSWER: Addressed.**

4. It should be also describe clearly what did the authors do to minimize and optimize the protein structure. I am very puzzling why did the author exploited computational server like SwissPDB to “optimize” the experimental crystal structure obtained from PDB?
**ANSWER: We minimized and optimized PDB structure because it has missing atoms and residues. Also it is crucial step for proper bond order in protein.**

5. The results from the binding site prediction to show all the putative active/allosteric sites should be included as a figure. The validation of the binding prediction is also important and should be added in the paper.

**ANSWER: Addressed.**

6. The ligand database is not clearly defined. It is unclear what computational or experimental approach the authors exploited to pre-screen the top 37 flavonoids before performing molecular docking with AutoDock Vina. The structures/structural illustration of all the compounds from the flavonoid library used in this work should be included as a supplementary material. Top 37 flavonoids with greatest binding should also be described structurally in the supplementary together with ADMET results.

**ANSWER: ADMET analysis of compounds is provided in supplementary data. 37 flavonoid compounds were chosen from different literature reviews.**

7. Line 116 “Toxicity Analysis” is a misleading title for a subsection because in fact the toxicity analysis is just part of ADMET analysis. The authors should make it clear.

**ANSWER: Addressed.**

8. Lead identification section is also not very clear of how did the author choose lead. Is there any particular experiment or analysis the author perform to exclude potentially false hit out from the results apart from looking at the calculated binding energy and the drug-likeness result of ADMET analysis.

**ANSWER: Researchers employed a range of metrics to meticulously assess potential inhibitors, including docking scores, interactions, logP coefficient, rotatable bonds, ring presence, PSA, Blood-Brain Barrier permeability, and Ames Toxicity. This multi-dimensional approach narrowed down the pool of inhibitors to a promising subset. Notably, the 2C Coxsackie B virus protein inhibitor stood out due to low binding affinities and strong lead-likeness scores. Positive interactions and compatibility with the target protein further supported its selection. The thorough use of diverse metrics led to identifying the 2C Coxsackie B virus protein inhibitor as a high-potential option for drug development.**

9. There is no need to add Ramachandran plot and the structure from PDB in the main figure. Figure 1 could be moved to supplementary information.

**ANSWER: Addressed.**

10. The structurally optimized model (perhaps superimposed with the PDB data) should be added in the figure and the pre-screening of the library should be at least mentioned. The figure to show binding prediction results should be added together with validation.

**ANSWER: Addressed in fig 1.**

11. Figure 2 there is no detail about bond length in the figure. Also this pocket should be shown together with the full structure.

**ANSWER: Addressed**

12.The Figure 3 that showed RMSD from simulations requires a lot of explanation. Is this one time simulation with no repeat? Normally, RMSD from all the repeat of MD simulation should be included in the figure. Also, is there any constraint used in the simulation? The authors should also discuss the fact that ligand RMSD shown in this figures could not displace beyond 1.5 A. Based on the pattern of the chart, it looks like there is a cutoff constraints at approximately 1.5 A?

**ANSWER: We used state of the art tool to perform a very long 300 ns simulation. All the parameters are mentioned in the manuscript. We did not use any cutoff to limit the RMSD of the ligand. This behavior of ligand RMSD is explained in the manuscript. For further investigation, trajectory files could be found at this link: https://drive.google.com/file/d/1hpx9TGYe2nA4z9VOhvwgrvsoBV7Y8UEM/view?usp=sharing.**

13. The rest of the figures could be improved. Many of them should be included not as main figures but as supplementary figures.
**ANSWER: Addressed**

14. All structures used in this study including protein, ligands, protein-ligand complexes should be deposited. The topology files used in trajectory analysis from MD simulations should be included as supplementary.

**ANSWER: Addressed**
